# Dietary Patterns, Nutritional Status and Inflammatory Biomarkers in Adolescents from the RPS Birth Cohort Consortium

**DOI:** 10.3390/nu15214640

**Published:** 2023-11-01

**Authors:** Eduarda Gomes Bogea, Maylla Luanna Barbosa Martins, Ana Karina Teixeira da Cunha França, Antônio Augusto Moura da Silva

**Affiliations:** Postgraduate Programme in Collective Health, Federal University of Maranhão, São Luís 65020-070, Brazil; mayllabmartins@gmail.com (M.L.B.M.); ana.franca@ufma.br (A.K.T.d.C.F.); aamouradasilva@gmail.com (A.A.M.d.S.)

**Keywords:** inflammation, food consumption, principal component analysis, nutritional status, teenagers

## Abstract

This study aimed to identify the dietary patterns (DPs) of adolescents and assess indicators of subclinical inflammation. It was a cross-sectional study aligned with the RPS cohort with data from São Luís, Maranhão, Brazil. We evaluated 511 adolescents between 18–19 years old. DPs were identified with a factor analysis of the principal components. Nutritional status was assessed with body mass index and body fat percentages. Hierarchical modeling was performed using a linear regression to estimate the beta coefficient (β) of the independent variables with the dependent variables interleukin-6 and high-sensitivity C-reactive protein (hs-CRP). Five DPs were identified: energy-dense, sugar-sweetened beverages and breakfast cereals, prudent, traditional Brazilian and alcoholic and energy beverages. Greater adherence to the prudent DP was associated with a lower concentration of interleukin-6 (β = −0.11; *p* value = 0.040). Greater adherence to the DP “traditional Brazilian” and “alcoholic and energy beverages” were associated with increased IL-6, mediated by the nutritional status. A higher BMI (β = 0.36; *p* value = <0.001) and %BF (β = 0.02; *p* value = 0.014) were associated with higher hs-CRP concentrations. The nutritional status and “prudent” pattern were associated with inflammatory biomarkers. These findings show that a higher consumption of fresh and minimally processed foods and the adequacy of the nutritional status are protective factors for the inflammatory process.

## 1. Introduction

Subclinical inflammation is characterized by increased levels of inflammatory markers produced by molecular mechanisms of innate and acquired immunity. This state of inflammation is associated with several non-transmissible diseases, such as diabetes, dyslipidemia and obesity, and identified as a pathogenic mechanism for the onset and progression of cardiovascular diseases (CVD) [1].

The inflammatory biomarkers C-reactive protein (CRP) and interleukin-6 (IL-6) were found to predict cardiovascular risk [2]. CRP is an acute-phase reagent protein produced by the liver with an important role in the atherosclerotic process and endothelial modulation [3]. Interleukin-6 is a pro-inflammatory cytokine that coordinates the immune response, acting as a signal for the release of other cytokines, such as interleukin-1 (IL-1) and tumor necrosis factor alpha (TNF-α) [4].

The accumulation of adipose tissue, commonly known as body fat, stands out among the factors that cause inflammation. Body fat is hormonally active and produces cytokines and acute-phase proteins that increase the production and circulation of factors related to inflammation in all age groups, including adolescents [5,6]. Current evidence also points to lifestyles, such as the level of physical activity [7] and eating habits [8], as inflammatory stimuli regardless of nutritional status.

One way of assessing eating habits is through the dietary pattern (DP) approach. This analysis can better describe the diet consumed by a population since it considers the complexity of the human diet and nutrient combinations [9]. This approach has been very useful to measure the risk for chronic diseases in relation to exposure to the diet of different populations, and it is effective in identifying associations between consumption, non-communicable diseases (NCDs) and pro-inflammatory biomarkers [10,11,12].

Bibiloni et al. [12] studied female adolescents in Spain and found that a greater adherence to the “Western” pattern was associated with lower plasma concentrations of adiponectin and higher levels of IL-6 while the “Mediterranean” pattern showed a higher plasma concentration of adiponectin.

Therefore, because of the scarcity of studies and the importance of more complex information on the food consumption of adolescents in addition to the possible relationship of food consumption with aspects, such as lifestyle and excess weight and subclinical inflammation, this study aimed to identify the main dietary patterns and evaluate the factors associated with inflammatory biomarkers.

## 2. Materials and Methods

### 2.1. Study Design

A cross-sectional study with data from the Brazilian cohort study ‘Lifelong Determinants of Obesity, Precursors of Chronic Diseases, Human Capital and Mental Health—RPS Birth Cohorts Consortium’ was conducted in the cities São Luís—Maranhão, Ribeirão Preto—São Paulo and Pelotas—Rio Grande do Sul. This study was only based on data from the cohort São Luís—Maranhão and was approved by the Research Ethics Committee of the University Hospital of the Federal University of Maranhão (No. 1,302,489).

### 2.2. Population and Sample

The perinatal study of the São Luís Cohort started at birth in ten public and private hospitals in the city from March 1997 to February 1998. Systematic sampling with proportional stratification was used according to the number of births in each hospital (1 in every 7 births). The sample tended to represent the births in the city since hospital births accounted for 96.3% of all births. Losses due to refusal or impossibility of locating the mother occurred in 5.8% of the total, with a final sample of 2443 hospital births.

The São Luís cohort was followed from 7 to 9 years of age (2005–2006) using a complex sampling design using the variable birth weight to define the sample required for the assessment at school age. The participation rate was 72.7% of the previous follow-up sample (673 participants).

**Figure 1:**
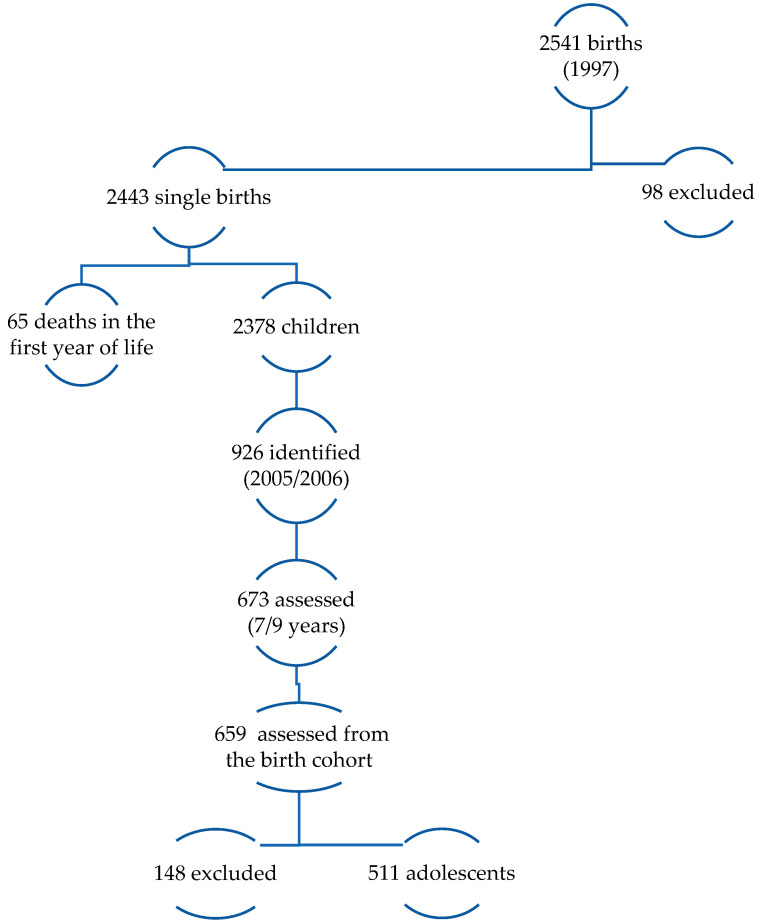
Sampling of the cohort of São Luís, Maranhão in the years 1997–98/2005–06/2016.

In the third phase of the cohort, location of participants was determined by searching enrollments of schools and universities, addresses and telephones recorded in the first and second phase of the cohort, military enlistment records for boys and social media. The searches identified 659 adolescents who agreed to participate and attended for data collection. This study considered the data from 511 participants since 106 had no C-reactive protein and interleukin-6 biomarkers evaluated and 42 had no anthropometric or food consumption data collected (Figure 1).

### 2.3. Data Collection

Data collection was carried out by trained interviewers. The food questionnaires were applied only by nutritionists because of their good practice in assessing food consumption. After identifying all the sample units, the adolescents were contacted to schedule for the exams and to respond to the general questionnaire and the FFQ food survey.

The general questionnaire was a standardized interview applied by the RPS cohort consortium, consisting of six sections (A–F). This study used data referring to “Family, housing and income”, the variables gender (female and male), Brazilian Criteria of Socioeconomic Classification (CEB) (A; B1 and B2; C1 and C2; D and E) and skin color (white, brown, black and yellow).

Food consumption was assessed with an electronic semi-quantitative FFQ regarding habitual consumption in the past 12 months, which was developed by Schneider et al. [13] and validated by Bogea et al. [14]. The daily consumption of food in grams or milliliters was converted into amount of macronutrients using the Brazilian Food Composition Table (TACO) [15], the Nutrient Database for Standard Reference (USDA) [16] or information available on the food labels. The analysis of food consumption data was performed using the software STATA 14.0.

Weight–height adequacy was assessed using body mass index (BMI), calculated by dividing a person’s weight (kg) by the square of height (m) and stratified by age and sex according to the World Health Organization for teenagers. Body composition was assessed with body fat percentage estimated by the air displacement plethysmography method using the COSMED Bod Pod^®^ Gold Standard analyzer (COSMED USA, Inc., Concord, CA, USA).

The physical activity level was measured by the 24 h Physical Activity Recall (PAR) adapted from the Self-Administered Physical Activity Checklist—SAPAC [17]. The level of physical activity was estimated by computing the metabolic equivalents of tasks (MET) per week and multiplying the related metabolic equivalent (MET) to the self-reported time spent in each activity. METs for each activity were compared against the Compendium of Physical Activities (CAF) [18]. The level of physical activity was classified according to the cutoff points of the International Physical Activity Questionnaires (IPAQ) expressed as MET/week: sedentary (0), low (1 to <600), moderate (600 to <3000) and high (≥3000) [19].

Only 40 mL of blood was collected from the cubital vein, which was centrifuged and stored in a freezer at −20 °C. Part of the blood that was not processed immediately was stored in freezers at −80 °C. Pro-inflammatory markers were analyzed using the Multiplex MAP Human Cytokine Kit technology, manufactured by Merck (Darmestadt, Germany), and were evaluated as continuous numerical variables. The immunological markers evaluated in this study were as follows: ultrasensitive C-reactive protein (hs-CRP) in ng/mL and IL-6 in pg/mL.

The hs-CRP is one of the most sensitive biomarkers for the inflammatory process and one of the most used in clinical practice, and it is easily analyzed by clinical analysis laboratories and affordable from a financial point of view. Investigating its behavior on this biomarker in relation to lifestyle (food consumption, smoking and physical activity), socioeconomic characteristics and nutritional status in a younger population, such as adolescents, brings greater clarity about its use in the evaluation of health by health professionals [20].

Interleukin-6 is a pro-inflammatory cytokine responsible for coordinating the immune response, as it is a signal of the inflammatory process. Activation of IL-6 receptors results in intracellular activation of the JAK/STAT (Janus kinase/signal transducers and activators of transcription) pathway with resulting production of other inflammatory cytokines, such as interleukin-1 (IL-1) and tumor necrosis factor alpha (TNF-α). Therefore, the use of this cytokine was prioritized [21,22].

### 2.4. Statistical Analysis

The dietary patterns were identified using the PCA method and varimax orthogonal rotation. The data suitability for the factor analysis was confirmed using the Kaiser–Meyer–Olkin (KMO) test, with KMO ≥ 0.60 [23] indicating the sampling was adequate. The number of retained factors was based on the criteria: components with eigenvalues greater than 1.5, Cattel graph (scree plot) and conceptual meaning of the identified patterns. Interpretation of each principal component was based on foods with factor loadings ≥ 0.30 or ≤−0.30, which was considered an important contribution to the pattern. Within a component, negative loads indicated an inverse association of the food item, and positive loads indicated a direct association [24].

Dietary patterns were labeled according to the nutritional composition of foods for each factor, and adolescents were given a score for each factor retained. The score of dietary patterns were categorized into tertiles, in which the upper tertile of the distribution represented greater adherence to the pattern.

The differences between adherence to dietary patterns and level of physical activity, nutritional status (BMI and %BF) and quantitative data on food consumption (total gross calories, macronutrients and micronutrients adjusted for energy) were assessed using the Chi-square test for categorical variables and the Kruskal–Wallis test for continuous variables, as they were not normally distributed. The measures of central tendency and variance used for continuous variables were median and interquartile range.

Hierarchical modeling was performed using linear regression to estimate the beta coefficient (β) of the independent variables with the dependent variables, IL-6 and hs-CRP, transformed into logarithms, as they were not normally distributed. In this analysis strategy, the introduction of variables took place in stages, starting with variables from the most distal levels and simultaneously introducing only variables from the same level. The effect of each variable on the outcome was interpreted as adjusted for variables belonging to hierarchically previous levels (more distal) and for the effects of variables located at the same level [25]. The choice of variables for each level took into account the intensity of determination at adjacent levels and at the stage (inflammation).

**Figure 2:**
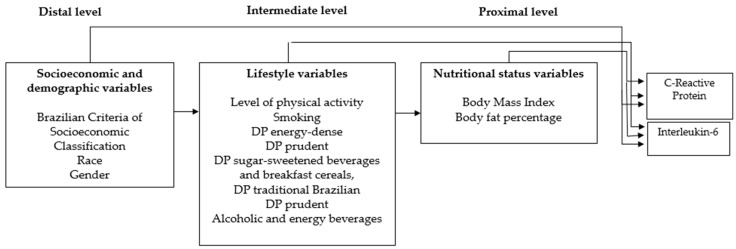
Description of independent variables according to hierarchical model, the RPS cohort, São Luís, MA, Brazil, 2016–2017.

Level constitution and ordering followed a previous theoretical model of determination and temporal precedence. Socioeconomic (CEB) and demographic variables (race and gender) were placed in the distal level, as they had less determination in relation to the outcome [26]. Lifestyle variables, such as the level of physical activity, smoking and food consumption, through dietary patterns were placed in the intermediate level [27,28]. Adiposity status variables (BMI and %BF) constituted the proximal level, as they presented greater determination in relation to the outcome [29] (Figure 2).

The variables with *p* < 0.05, at any level of the theoretical model, were considered significant and formed the group of variables of the next level. This procedure repeated until the final level. The interpretation of the β coefficient was carried out at the level to which the variable belonged. Confidence intervals (95% CI) were estimated. Significance level was set at 5%. Statistical analyses were performed using the STATA program, version 12.0.

## 3. Results

Of the 511 participants evaluated, 61.9% were male, 64.9% reported brown skin color and mean age was 18.1 ± 0.2 years. Most adolescents were from socioeconomic class B (47.2%) and C (42.3%). The lifestyle evaluation showed that 34.4% were sedentary and 90.8% non-smokers. The prevalence of being overweight, according to BMI, was 18.8%, with 12.9% of participants overweight and 5.9% obese.

The test assessing correlations between food items and the adequacy of using a factor analysis to identify DP was satisfactory and appropriate for PCA (KMO = 0.723). The scree plot and eigenvalues ≥ 1.5 determined the extraction of five factors. Dietary patterns were defined as follows: energy-dense, sugar-sweetened beverages and breakfast cereals, prudent, traditional Brazilian and alcoholic and energy beverages. The extraction of these components explained 36.1% of the total variance after factor rotation, with the highest proportion of the total variance retained in the “energy-dense” pattern (9.4%), which best represented the dietary intake of the sample (Table 1).

Adolescents who adhered more to the “energy-dense” DP were more active and had a higher intake of lipids, saturated fat, omega 6, niacin and sodium and a lower intake of carbohydrates, protein, fiber and vitamin C. The “sugar-sweetened beverages and breakfast cereals” DP provided a higher intake of carbohydrates, fiber, calcium and vitamin C and a lower intake of protein, lipids, omega 6, niacin, sodium and cholesterol. The “prudent” DP showed greater adherence by active adolescents with a lower %BF. For the intake of nutrients, there was a higher consumption of protein, fiber, iron, niacin, vitamin C, omega 3 and cholesterol and a lower intake of carbohydrates, lipid, saturated fat and sodium (Table 2).

Active adolescents had greater adherence to the “traditional Brazilian” DP and showed no excess weight and lower %BF. For the intake of nutrients, they had a lower consumption of lipids, saturated fat, niacin, vitamin C and cholesterol. Adolescents who adhered more to the “alcoholic and energy beverages” DP were more active, had a higher intake of carbohydrates and vitamin C and a lower intake of protein, lipids, saturated fat, fiber, calcium, iron, thiamine and cholesterol (Table 3).

No association was found between socioeconomic and demographic variables with IL-6. After adjustment, the hierarchical analysis showed that the “prudent”, “traditional Brazilian” and “alcoholic and energy beverages” DPs were significant predictors for IL-6, with the dietary scores of “prudent” (β= −0.11; *p* value = 0.040) and “traditional Brazilian” (β = −0.12; *p* value= 0.027) being inversely related to plasma concentrations of IL-6. The dietary score of the PA “alcoholic and energy beverages” (β = 0.11; *p* value = 0.041) was directly related to the plasma concentrations of IL-6. In the analysis of the proximal level, only the “prudent” DP remained significant, indicating that the associations of the “traditional Brazilian” and “alcoholic and energy beverages” DPs are mediated by the nutritional status (Table 4).

No association was found between the variables of socioeconomic, demographic and lifestyle characteristics and hs-CRP. The variables of nutritional status were directly associated with hs-CRP, both BMI (β = 0.36; *p* value = <0.001) and %BF (β= 0.02; *p* value = 0.014) (Table 5).

## 4. Discussion

Five dietary patterns were identified: energy-dense, sugar-sweetened beverages and breakfast cereal, prudent, traditional Brazilian, and alcoholic and energy beverages. These factors explained 36.6% of the variance, and the “energy-dense” DP made the greatest contribution to the proportional variance. Subclinical inflammation assessed by IL-6 showed a direct association of the “prudent” DP as a protective factor. The DPs “traditional Brazilian” and “alcoholic and energy beverages” were associated with IL-6 concentrations, mediated by the nutritional status. The hs-CRP concentrations were directly associated with excess weight and body fat.

Generally, DP studies use PCA extract from two to four patterns, and these patterns are represented by the consumption of unhealthy foods, healthy foods and traditional foods in the studied site. Gutiérrez-Pliego et al. [30] assessed adolescents aged 14 to 15 years in Mexico and identified the patterns ‘Westernized’, ‘High animal protein and fat’ and ‘Prudent’. A study by Bibiloni et al. [12] carried out in Ireland with adolescents aged 12 to 17 years identified the ‘Western’ and ‘Mediterranean’ patterns. Pinho [31] carried out a study in Florianópolis-SC with adolescents between 11 and 14 years old, was the only one that used the same methodology and extracted five patterns, named ‘Obesogenic’, ‘Coffee and dairy products’, ‘Traditional Brazilian meal’, ‘Fruits and vegetables’ and ‘Bread and chocolate milk’.

In the present study, the “energy-dense” pattern made the highest contribution to the total variance and best represented the diet of this population. The “energy-dense” DP consists mainly of ultra-processed foods, with significantly higher amounts of saturated fat, omega-6, refined sugar and sodium and significantly lower amounts of carbohydrates, protein, fiber and vitamin C. The foods present in this pattern are characterized by high energy density.

The literature points out that the consumption of “energy-dense” foods and their nutrients are associated with a greater health risk [32], including mental health problems [33].

The relationship between increased consumption of these foods and the prevalence of various diseases is clear, necessitating greater government interventions to control the sale, dissemination and consumption of these products. These interventions could include higher taxation of fast food and sugary drinks, media and packaging controls appealing to children and adolescents and clearer labels on their nutritional composition.

However, no difference was found in the nutritional status of the adolescents evaluated between the tertiles of the consumption of the “energy-dense” pattern, and there was no association between this DP and the IL-6 and hs-CRP biomarkers. Possible explanations for this finding are underreporting the consumption of these foods by overweight individuals [24,34] and the fact that adolescents with greater adherence to this pattern were physically active.

The DP “sugar-sweetened beverages and breakfast cereals” consisted of natural fruit juices and milk, table sugar and breakfast cereals, with significantly higher amounts of carbohydrates, fiber, calcium and vitamin C and lower amounts of lipids, sodium and cholesterol. Despite the consumption of sugar and breakfast cereals that are rich in simple carbohydrates, this DP includes natural fruit juice and milk, foods that are sources of important nutrients. This DP showed no difference between the consumption tertiles and nutritional status and physical activity level, and it was not associated with inflammatory biomarkers.

The “prudent” DP consisted predominantly of fresh or minimally processed foods, rich in antioxidants, micronutrients and fiber. This pattern stands out for providing the greatest amount of the macronutrient protein. Adolescents with greater adherence to this dietary pattern were more active and showed the lowest %BF. The adherence to the “prudent” DP was associated with a decrease in IL-6 concentrations, indicating that it protects against subclinical inflammation, regardless of the adolescent’s nutritional status and level of physical activity.

This protection can be explained by the high amount of dietary fiber, which plays a crucial role in the intestinal microbiota [35]. The health of intestinal microbiota has a systemic impact and is directly related to the immune system as well as to the inflammatory process [36]. The most recognized biological plausibility for this process is endotoxemia, which is defined as an increase in the endotoxin lipopolysaccharide (LPS) in the blood stream. LPSs are part of the cell wall composition of gram-negative bacteria that bind to the Toll-like receptor (TLR-4) present in the plasma membrane of cells, boosting several signaling pathways that lead to inflammation through the activation of the genes that encode the proteins involved, such as TNF-α and IL-6 [37].

In addition, the antioxidant components of plant foods contribute to their anti-inflammatory effect, and their intake is inversely related to concentrations of pro-inflammatory cytokines, e.g., IL-6 [38,39]. Interleukin-6 is a pro-inflammatory cytokine responsible for coordinating the immune response, as it is a signal of the inflammatory process. The activation of IL-6 receptors provoke in intracellular activation of the JAK/STAT (Janus kinase/signal transducers and activators of transcription) pathway with resulting production of other inflammatory cytokines, such as interleukin-1 (IL-1) [21,22]. Thus, it is claimed that the low adherence to the “prudent” DP alters the intestinal microbiota, signaling an immune response and triggering the inflammatory process.

In general, there are few actions and advertisements promoting the consumption of fresh foods specifically targeting the adolescent population while the food industry heavily invests in promoting its processed and ultra-processed products. Therefore, it is necessary for governments to invest more in effective public health education policies aimed at increasing the consumption of fresh foods. This includes interventions in school meals and the use of media to promote a more natural diet rich in vegetables, fresh fruits and meats.

The fourth DP extracted, named as “traditional Brazilian”, consisted of foods traditionally consumed by the Brazilian population and financially accessible, being part of the food culture of Brazil (rice and beans) and the northeast region of the country (cassava flour). Foods in this pattern are commonly consumed in the main meals, such as breakfast and lunch. From a nutritional point of view, this pattern has higher amounts of carbohydrates due to rice, bread and flour and lower amounts of lipids, saturated fat and cholesterol, even considering the content of oils and fats.

The “traditional Brazilian” DP was inversely associated with IL-6; thus, it is considered a protective factor for subclinical inflammation. However, in the proximal analysis, adjusted for nutritional status, it was found that this association was not maintained, showing an indirect effect of DP on IL-6, mediated by BMI and %BF. It is noteworthy that adolescents with great adherence to the “traditional Brazilian” DP had a lower prevalence of being overweight and lower %BF values.

Greater adherence to the “alcoholic and energy beverages” DP was associated with higher amounts of carbohydrates and vitamin C. The high amount of carbohydrates is related to the manufacturing of alcoholic beverages, which are made from the fermentation of sugars contained in fruits and vegetables, as it is high in caloric density (7 kcal/mL) and poor in nutrients. The study highlights the extraction of this dietary pattern in adolescents, in which alcoholic and energy beverages represent their eating habits. Other studies with adolescents did not identify this DP [30,31,40].

The role of alcohol in inflammation is controversial since high and chronic consumption causes inflammation [41], whereas stable and moderate consumption is protective [42]. Despite divergences in the literature about the consequences of drinking alcohol in low doses, at the beginning of 2023, the World Health Organization announced that ‘No level os alcohol consumption is safe whwn it comes to human health’ [43]. In this study, the positive association of this DP with subclinical inflammation, as assessed by IL-6, was mediated by the nutritional status.

A systematic review of the literature pointed out that in 81.0% of the studies, obesity-related biomarkers were not associated with dietary patterns [10], a result different from that found in the study, in the which adherence to the “prudent” DP was associated with a decrease in IL-6 concentrations. Similar to the present study, Bibiloni et al. [12] found association between IL-6 and the DP, with a higher concentration of this biomarker in female adolescents who had more adherence to the Western DP.

The hs-CRP was not associated with socioeconomic, demographic and lifestyle variables, but a direct association was found between this marker and nutritional status, both by BMI and by %BF. This finding shows a direct association between inadequate nutritional status and inflammation, regardless of other factors. This response may occur because adipose tissue is an active organ and, when in excess, releases several adipokines that, directly or indirectly, increase the production and circulation of inflammation factors [5]. The fact that CRP is a protein produced by the liver and plays an important role in the metabolic response makes it more sensitive to the presence of adipose tissue [1,2,3].

It is important that the increase in CRP precedes the appearance of cardiovascular diseases and diabetes. Therefore, increased CRP in overweight individuals as well as in adolescents is considered a relevant risk for such outcomes [3,44]. A meta-analysis study evaluated the intima-medial thickness of arteries in adolescents and found significant differences between the obese and non-obese groups, with higher values in the obese group, which presented a higher risk for coronary artery disease [45]. Once again, the study by Bibiloni et al. [12] found results similar to those of the present study, with a direct association between CRP and nutritional status in adolescents assessed by BMI and waist-to-height ratio.

Some limitations of this study can be highlighted. First, the nature of the study, as it is cross-sectional, does not allow for inferences to be made that involve the temporality of the effect, as the effect of the diet on outcomes considered chronic is better visualized in longitudinal studies. However, it is worth highlighting that we used an FFQ that sought to assess the frequency of food consumption in the last 12 months, which is more likely to reflect a habitual diet in that period of time prior to the collection of inflammatory markers. Second, using the FFQ as a standard for assessing food consumption can lead to some limitations resulting from the overestimation or underestimation of certain foods, which results from the effect of memory and food groupings.

The use of early inflammatory biomarkers hs-CRP and IL-6 in adolescents stands out as a strength, particularly IL-6, as it is a pro-inflammatory cytokine responsible for coordinating the immune response and signaling the inflammatory process. The use of a more robust methodology in assessing food consumption through dietary patterns is also a differentiator of the study, as it better expresses the diet consumed by a given population and reflects the variability inherent in food consumption. Furthermore, body composition is assessed using air displacement plethysmography, which is considered a more accurate method for measuring body adiposity.

## 5. Conclusions

The extraction of dietary patterns in adolescents shows a higher consumption of ultra-processed foods high in simple sugars while only the “prudent” DP represents a healthy diet. Inflammatory biomarkers were associated with nutritional status and with the “prudent” DP, indicating that adolescents with a higher BMI and %BF had higher hs-CRP concentrations and adolescents with greater adherence to the prudent DP had lower IL-6 concentrations. These findings show that a higher consumption of fresh and minimally processed foods, corresponding to the “prudent” DP, and the adequacy of the nutritional status are protective factors for the inflammatory process and, consequently, for metabolic and cardiovascular outcomes.

## Figures and Tables

**Table 1:** Distribution of factor loadings of the main dietary patterns identified in adolescents from the RPS cohort, São Luís, MA, Brazil, 2016–2017.

Food Group		Dietary Pattern			
	Energy-Dense	Sugar Sweetened Beverages and Cereal Breakfast	Prudent	Traditional Brazilian	Alcoholic and Energy Beverages
Junk food	0.789				
Sweets	0.642				
Cakes and cookies	0.614				
Processed meats	0.608				
Sauces	0.551				
Soda/Industrialized juice	0.430				
Canned food	0.381				
Natural Juice		0.803			
Added sugar		0.785			
Breakfast cereals		0.488			
Milk and derivatives		0.401			
Fish			0.647		
Vegetables			0.598		
Red meat and offal			0.579		
Chicken, birds			0.473		
Nuts			0.441		
Fruit			0.406		
Eggs			0.380		
Tapioca/couscous			0.346		
Tubers			0.322		
Rice				0.578	
Breads				0.561	
Fats				0.541	
Coffe				0.533	
Beans				0.385	
Flours				0.363	
Energy Beverages					0.716
Alcoholic Beverages					0.600
Guarana powder					0.339
Number of items	7	4	9	6	3
Proportional Variance	9.42%	7.52%	7.24%	6.76%	5.12%
Accumulated Variance	9.42%	16.94%	24.18%	30.94%	36.06%
KMO * coefficient	0.723				

* Kaiser–Meyer–Olkin.

**Table 2:** Physical activity, nutritional status and estimated dietary intake by tertiles of dietary patterns in adolescents from the RPS cohort, São Luís, MA, Brazil, 2016–2017.

	Energy-Dense			Sugar-Sweetened Beverages and Breakfast Cereals			Prudent		
	T1	T3	*p* Value	T1	T3	*p* Value	T1	T3	*p* Valor
Physical activity (%)			0.048 *			0.137			0.002 *
Sedentary	38.9	26.9		38.9	29.1		40.0	23.4	
Active	30.3	37.0		30.3	35.1		30.3	38.4	
Nutritional Status (BMI) ^1^			0.113			0.459			0.075
Without overweight	31.5	31.0		32.2	34.1		35.6	32.7	
Overweight	41.7	32.9		38.5	29.2		24.0	35.4	
Body fat (%)	18.3 (10.6–28.5)	16.6 (8.4–25.5)	0.119	16.6 (8.6–27.3)	16.8 (8.4–25.1)	0.602	18.1 (8.5–25.8)	15.7 (7.9 –24.0)	0.035 *
Dietary Intake									
Calories (kcal/day)	2082.7 (1539.5–2768.0)	3962.8 (3235.4–5244.3)	<0.001 *	2576.9 (1787.0–3661.2)	3661.2 (2671.1–4784.3)	<0.001 *	2575.2 (1671.6–3564.3)	3508.5 (2754.5–4716.1)	<0.001 *
Carbohydrates (g/day)	460.6 (423.6–486.0)	431.5 (401.0–459.6)	<0.001 *	427.7 (400.8–459.3)	455.7 (427.4–481.3)	<0.001 *	448.7 (418.0–73.6)	431.5 (403.5–62.2)	0.008 *
Protein (g/day)	100.7 (85.4–119.2)	96.1 (83.7–110.0)	0.040 *	103.7 (90.4–123.3)	93.4 (82.0–109.8)	0.001 *	89.6 (79.3–99.5)	114.5 (97.1–131.5)	<0.001 *
Lipid (g/day)	66.3 (57.2–75.9)	81.2 (70.8–92.0)	<0.001 *	77.9 (68.2–91.6)	69.8 (61.0–78.3)	<0.001 *	77.6 (66.7–90.1)	72.0 (61.6–81.6)	0.003 *
Saturated fat (g/day)	24.5 (20.5–30.0)	30.3 (26.1–34.8)	<0.001 *	28.2 (24.0–32.6)	26.9 (22.2–30.7)	0.076	29.1 (25.7–24.5)	25.9 (22.9–30.3)	<0.001 *
Omega 6 (g/day)	7.2 (5.7–9.3)	8.8 (7.2–11.0)	<0.001 *	8.8 (7.0–11.0)	7.3 (5.6–9.0)	<0.001 *	8.2 (6.4–10.2)	7.6 (6.3–9.7)	0.191
Omega 3 (g/day)	0.8 (0.7–1.0)	0.8 (0.7–1.1)	0.752	0.9 (0.7–1.1)	0.8 (0.6–1.0)	0.055	0.8 (0.6–1.0)	0.9 (0.7–1.1)	0.019 *
Fiber (g/day)	80.1 (64.9–101.6)	66.2 (56.5–79.9)	<0.001 *	65.9 (56.6–79.9)	76.4 (65.3–94.2)	<0.001 *	65.4 (55.1–79.9)	76.8 (64.8–95.1)	<0.001 *
Calcium (mg/day)	737.1 (528.8–932.2)	688.8 (565.0–863.1)	0.170	626.2 (495.2–772.1)	837.4 (649.7–1013.4)	<0.001 *	765.5 (573.2–980.2)	709.3 (569.9–876.6)	0.080
Iron (mg/day)	12.6 (10.8–15.1)	13.0 (11.1–15.0)	0.887	13.2 (11.1–15.5)	12.6 (10.9–14.7)	0.460	12.0 (10.2–14.5)	13.9 (11.6–16.7)	<0.001 *
Thiamine (mg/day)	1.4 (1.2–1.7)	1.5 (1.3–1.7)	0.675	1.4 (1.2–1.7)	1.5 (1.3–1.8)	0.075	1.4 (1.2–1.7)	1.5 (1.4–1.7)	0.076
Niacin (mg/day)	15.0 (11.4–18.9)	17.1 (13.3–21.4)	0.001 *	17.2 (13.4–21.2)	15.2 (12.0–18.7)	0.004 *	14.3 (11.7–17.7)	18.2 (15.1–18.2)	<0.001 *
Vitamin C (mg/day)	132.7 (86.0–224.1)	114.4 (63.0–167.6)	0.001 *	85.4 (51.5–135.0)	164.5 (112.7–238.5)	<0.001 *	97.0 (61.2–149.5)	144.0 (92.2–213.0)	<0.001
Sodium (mg/day)	1716.3 (1458.6–2026.5)	2084.7 (1823.4–2506.0)	<0.001 *	2043.2 (1681.0–1510.0)	1807.3 (1562.8–2140.8)	<0.001 *	2100.7 (1681.0–2603.5)	1811.3 (1547.3–2089.3)	<0.001
Cholesterol (mg/day)	377.6 (275.8–473.8)	374.5 (300.5–511.4)	0.563	418.5 (309.8–560.8)	343.6 (261.8–466.1)	<0.001 *	310.4 (251.5–378.8)	446.0 (343.5–574.5)	<0.001

^1^ Body Mass Index; * *p* value < 0.05; Kruskal–Wallis test to assess the differences between consumption tertiles of each dietary pattern.

**Table 3:** Physical activity, nutritional status and estimated dietary intake by tertiles of dietary patterns in adolescents from the RPS cohort, São Luís, MA, Brazil, 2016–2017.

	Traditional Brazilian			Alcoholic and Energy Beverages		
	T1	T3	*p* Value	T1	T3	*p* Valor
Physical activity (%)			0.006 *			0.001 *
Sedentary	41.1	24.6		36.0	22.9	
Active	29.7	37.4		31.8	38.7	
Nutritional Status (BMI) ^1^			0.010 *			0.147
Without overweight	30.8	35.8		35.3	32.0	
Overweight	44.8	21.9		25.0	38.5	
Body fat (%)	20.6 (12.4–28.5)	12.9 (6.9–20.7)	<0.001 *	17.8 (8.6–25.0)	15.4 (8.4–24.8)	0.332
Dietary Intake						
Calories (kcal/day)	2128.9 (1601.2–3318.0)	3526.4 (2899.2–4768.9)	<0.001 *	2971.1 (2161.4–4091.1)	3319.5 (2414.8–4027.9)	<0.001 *
Carbohydrates (g/day)	435.1 (404.4–462.9)	448.0 (419.4–475.3)	0.037	428.0 (398.8–453.0)	458.8 (20.7–481.3)	<0.001 *
Protein (g/day)	98.0 (85.4–116.4)	95.3 (84.7–112.3)	0.869	107.6 (89.9–125.2)	92.6 (80.7–105.5)	<0.001 *
Lipid (g/day)	75.4 (66.3–90.7)	71.9 (61.9–81.3)	0.021 *	75.8 (67.9–89.3)	70.8 (61.0–82.7)	0.004 *
Saturated fat (g/day)	29.6 (25.1–33.8)	26.7 (22.0–30.4)	0.001 *	29.2 (25.8–33.4)	26.8 (22.0–31.1)	0.001 *
Omega 6 (g/day)	8.4 (6.5–10.6)	7.9 (6.1–10.0)	0.358	7.8 (6.6–10.7)	8.0 (6.0–10.0)	0.687
Omega 3 (g/day)	0.8 (0.6–1.0)	0.9 (0.7–1.0)	0.360	0.9 (0.7–1.0)	0.8 (0.6–1.0)	0.100
Fiber (g/day)	73.3 (59.6–86.9)	71.5 (60.6–88.2)	0.831	74.5 (62.0–96.9)	68.6 (58.5–81.8)	0.053
Calcium (mg/day)	708.2 (545.2–889.3)	733.4 (586.1–888.3)	0.352	812.3(629.6–1036.3)	647.3 (519.0–785.6)	<0.001 *
Iron (mg/day)	13.3 (11.4–15.5)	12.5 (10.4–14.8)	0.053	13.9 (11.6–16.1)	12.2 (10.7–13.8)	<0.001 *
Thiamine (mg/day)	1.5 (1.3–1.8)	1.5 (1.3–1.7)	0.312	1.6 (1.4–1.8)	1.4 (1.2–1.6)	<0.001 *
Niacin (mg/day)	17.1 (14.2–22.0)	15.0 (12.1–18.3)	<0.001 *	15.9 (12.8–20.1)	16.4 (12.6–21.4)	0.056
Vitamin C (mg/day)	157.9 (103.1–245.6)	98.8 (59.4–163.9)	<0.001 *	107.5 (71.3–159.4)	139.3 (84.1–220.5)	0.011 *
Sodium (mg/day)	1875.9 (1569.1–2184.7)	1984.0 (1689.1–2320.2)	0.178	2025.3 (1705.3–2345.7)	1847.6 (1584.0–2297.6)	0.061
Cholesterol (mg/day)	375.8 (300.5–539.9)	338.6 (263.6–457.8)	0.021 *	410.8 (321.0–535.9)	350.0 (261.8–455.9)	<0.001 *

^1^ Body Mass Index; * *p* value < 0.05; Kruskal–Wallis test to assess the differences between consumption tertiles of each dietary pattern.

**Table 4:** Linear regression analysis of the association between interleukin-6 and socioeconomic, demographic, lifestyle and nutritional status variables in adolescents from the RPS cohort, São Luís, MA, Brazil, 2016–2017.

		Interleukin 6					
		Non-Adjusted			Adjusted		
		Beta	IC ^1^ 95%	*p* Value	Beta	IC ^1^ 95%	*p* Value
Distal level *: variables	Color	0.01	−0.12–0.15	0.883	0.30	−0.11–0.18	0.690
socioeconomic and	CEB ^2^	−0.09	−0.26–0.08	0.283	−0.11	−0.29–0.18	0.220
demographics	Gender	0.13	−0.09–0.34	0.250	0.09	−0.15–0.33	0.468
	Energy Dense	−0.05	−0.15–0.06	0.376	−0.04	−0.15–0.06	0.445
	Sugar sweetened beverages and cereal breakfast	−0.05	−0.15–0.06	0.383	−0.04	−0.15–0.06	0.4183
	Prudent	−0.12	−0.22–−0.01	0.029 ^¥^	−0.11	−0.22–−0.01	0.040 ^¥^
Intermediate level **: lifestyle variables	Traditional Brazilian	−0.12	−0.23–−0.02	0.020 ^¥^	−0.12	−0.22–−0.01	0.027 ^¥^
	Alcoholic and energy beverages	0.09	−0.13–0.20	0.085	0.11	0.01–0.22	0.041 ^¥^
	smoking	−0.21	−0.58–0.15	0.250	−0.25	−0.62–0.12	0.193
	Physical activity	−0.04	−0.13–0.04	0.345	−0.02	−0.11–0.07	0.696
Proximal level ***: adiposity status variables	BMI ^3^	0.12	0.00–0.23	0.042	0.03	−0.11–0.17	0.670
	Body Fat	0.01	0.00–0.02	0.006 ^¥^	0.01	0.00–0.21	0.077

^1^ Confidence Interval; ^2^ Brazilian Economic Classification; ^3^ Body Mass Index; ^¥^ *p* value < 0.05. * Distal level: adjustment made for the variables of this level. ** Intermediate level: adjustment made for the variables of this level and for the significant variables (*p* < 0.05) of the previous level (distal level). *** Proximal level: adjustment made for the variables of this level and for the significant variables (*p* < 0.05) of the previous levels (distal level and intermediate level).

**Table 5:** Linear regression analysis of the association between C-reactive protein and socioeconomic, demographic, lifestyle and nutritional status variables in adolescents from the RPS cohort, São Luís, MA, Brazil, 2016–2017.

		C-Reactive Protein					
		Non-Adjusted			Adjusted		
		Beta	IC ¹ 95%	*p* Value	Beta	IC 95%	*p* Value
Distal level *: variables	Color	−0.02	−0.18–0.13	0.775	−0.02	−0.19–0.15	0.809
Socioeconomic and demographics	CEB ^2^	−0.01	−0.20–0.18	0.926	−0.01	−0.21–0.19	0.938
	Gender	0.06	−0.19–0.31	0.635	0.02	−0.25–0.30	0.850
	Energy-Dense	0.05	−0.07–0.17	0.420	0.04	−0.09–0.16	0.569
	Sugar sweetened beverages and cereal breakfast	0.04	−0.08–0.16	0.493	0.03	−0.09–0.15	0.625
	Prudent	0.03	−0.09– 0.15	0.590	0.02	−0.14–−0.11	0.784
Intermediate level **: lifestyle variables	Traditional Brazilian	−0.03	−0.15–0.09	0.607	−0.03	−0.15–−0.09	0.640
	Alcoholic and energy beverages	0.11	−0.01–0.24	0.062	0.10	−0.03–0.23	0.130
	smoking	0.05	−0.37–0.46	0.825	−0.06	−0.49–0.37	0.791
	Physical activity	0.08	−0.02–0.18	0.107	−0.06	−0.04–0.17	0.255
Proximal level ***: adiposity status variables	BMI ^3^	0.48	0.35–0.60	<0.001 ^¥^	0.36	0.21–0.51	<0.001 ^¥^
	Body Fat	0.03	0.02–0.04	<0.001 ^¥^	0.02	0.00–0.03	0.014 ^¥^

^1^ Confidence Interval; ^2^ Brazilian Economic Classification; ^3^ Body Mass Index; ^¥^ *p* value < 0.05. * Distal level: adjustment made for the variables of this level. ** Intermediate level: adjustment made for the variables of this level and for the significant variables (*p* < 0.05) of the previous level (distal level). *** Proximal level: adjustment made for the variables of this level and for the significant variables (*p* < 0.05) of the previous levels (distal level and Intermediate level).

## Data Availability

The data that support the findings of this study are available by e-mail: rosangela.flb@ufma.br, but restrictions apply to the availability of these data, which were used under license for the current study and so are not publicly available. Data are, however, available from the authors upon reasonable request and with permission from Rosangela Fernandes Lucena Batista.
